# The impact of *Helicobacter pylori* and intestinal helminth infections on gastric adenocarcinoma and inflammatory bowel disease in Sub-Saharan Africa

**DOI:** 10.3389/fmed.2022.1013779

**Published:** 2022-12-09

**Authors:** Mashiko Setshedi, Gillian Watermeyer

**Affiliations:** Division of Gastroenterology, Department of Medicine, Groote Schuur Hospital, University of Cape Town, Cape Town, South Africa

**Keywords:** *Helicobacter pylori*, inflammatory bowel disease, gastric cancer, Africa, helminths

## Abstract

Gastric adenocarcinoma (GCA) is the 5th leading cancer globally with an estimated 1.1 million cases reported in 2020. Ninety percent of non-cardia GCAs are attributable to *Helicobacter pylori* (*H. pylori*), the most prevalent bacterial infection globally. Rates of *H. pylori* infection are highest in Sub-Saharan Africa (SSA), yet surprisingly low numbers of GCAs are reported in the region. A similar phenomenon is seen with the inflammatory bowel diseases (IBD), Crohn’s disease, and ulcerative colitis. These disorders have risen dramatically over the past century in high income countries across the globe, with sharp increases noted more recently in newly industrialized regions. In contrast IBD is rare in most regions in SSA. For both diseases this may reflect under-reporting or limited access to diagnostic modalities, but an alternative explanation is the high burden of infection with gastrointestinal parasites endemic to SSA which may attenuate the risk of developing GCA and IBD. In this mini review we discuss the complex interplay between these microorganisms, GCA, and IBD, as well as a possible protective role of *H. pylori* and the development of IBD.

## Introduction

Gastric adenocarcinoma (GCA) is the 5th leading cancer globally and the 4th leading cause of cancer death worldwide. Data from the GLOBOCAN 2020 database estimated that 1.1 million cases of gastric cancer were diagnosed worldwide in 2020 with considerable geographic variation (1). Eastern Asian regions showed the highest incidence rates in both males and females (age standardized rates of 32.5 and 13.2 per 100,000, respectively). About two thirds of all cases occur in men. Ninety percent of non-cardia GCAs are attributable to *Helicobacter pylori* (*H. pylori*) which is the most frequently encountered bacterial infection worldwide (2). Although data on *H. pylori* prevalence in SSA is limited, a 2016 systematic review with meta-analysis including 184 studies and evaluating the prevalence of *H. pylori* infection worldwide, found that Africa had the highest rate with a prevalence of 70.1% (2). In another meta-analysis Nigeria was found to have the highest prevalence globally with a rate of 89.7% (3). Despite this the number of reported cases of GCA in SSA is surprisingly low. The GLOBOCAN data base reported age standardized GCA rates between 4.6 to 4.9 and 2.4 to 4.2 per 100,000 in men and woman, respectively from SSA (1).

A similar phenomenon is seen with the inflammatory bowel diseases (IBD), Crohn’s disease (CD), and ulcerative colitis (UC). These are chronic inflammatory disorders of the gastrointestinal tract, characterized by periods of remission and intercurrent flares of disease activity. IBD is thought to result from complex interactions between environmental factors (such as tobacco and diets high in animal fats or processed foods), in a genetically susceptible host, resulting in an aberrant immune response to the intestinal flora (4, 5). These disorders have risen dramatically over the past century in high income countries across the globe, with sharp increases noted more recently in newly industrialized regions (6, 7). In contrast IBD is rare in most regions in SSA (8–10).

For both diseases the relative paucity of cases may reflect under-reporting or limited access to diagnostic modalities, but an alternative explanation is the high burden of infection with gastrointestinal parasites endemic to SSA which may attenuate the risk of developing GCA and IBD (11, 12).

Soil-transmitted helminth (STH) infections are widespread in SSA as a consequence of poor socioeconomic status and environmental sanitation. The most common STHs are *Trichuris trichiura*, *Ascaris lumbricoides*, and the hookworms (*Necator americanus* and *Ancylostoma duodenale).* Infection with these STHs modulates host immune responses, induces systematic immune tolerance, and suppresses inflammatory responses (11, 12).

## Gastric cancer in SSA

### *H. pylori* and gastric cancer

The most established risk factor for intestinal GCA is *H. pylori*, with an attributable risk of 75–88% (13, 14). The mechanism involves an interplay between the host, gastric microenvironment, and bacterial virulence factors (15). These strain-specific virulence factors, cytotoxin-associated gene A (CagA) and vacuolating cytotoxin A (VacA), are not only involved in the induction and maintenance of inflammatory responses, but enable the colonization and survival of *H. pylori* within the gastric mucosa, leading to further immune escape, the induction of premalignant alterations and eventually GCA (15, 16). Histologically, based on the Lauren classification, there are four types of GCA: the intestinal type (by far the commonest), diffuse type, mixed and indeterminate, or unclassifiable subtypes (17). Intestinal GCA specifically, progresses in the stomach through the Correa sequence, with a stepwise linear progression from chronic gastritis, gastric atrophy, gastric intestinal metaplasia, dysplasia and finally, invasive cancer (18). Lending credence to its importance, this original model was amended to involve the role of *H. pylori* as the prime causative environmental agent (19).

### The “African enigma” and role of helminths

Published literature, however, suggest that GCA appears to be rare, or not as high as would be expected in Africa. This is in the context that more than two thirds of the population in SSA have *H. pylori* infection (20). This dissociation has been famously termed the “African enigma” by Holcombe et al. (21) on the basis that based on data the rate of occurrence of GCA in some regions of Africa is roughly between a low 2 and 3%. These low rates he argues, may be due to acquisition of infection in childhood, which confers a less pathogenic inflammatory phenotype, the low rate of intestinal metaplasia (a precursor to GCA), and protective environmental factors such as low rates of smoking and a nutritious diet of vegetables in Africa. Further arguments proposed to support this theory by others include host factors and the high burden of parasitic infections which may bias the inflammatory cytokine response to a Th2 type (22). The latter argument was originally based on animal studies; the first showed that co-infection with nematodes in mice with *H. pylori* protects against gastric atrophy (23). It is reported that concurrent enteric helminth infection modulates inflammation and gastric immune responses and reduces *H. pylori*-induced gastric atrophy. This is mediated by downregulation of TNFα and IL1β, and upregulation of IL4 and IL10 (23). In a later study, these findings were corroborated in *H. pylori* infected transgenic mice, where co-infection with helminths resulted in similar degrees of inflammation, but reduced gastric atrophy (*p* < 0.04) and dysplasia (*p* < 0.02) (24). Few studies have been conducted in the adult human population, with no studies in SSA. Notwithstanding, a recent study performed in Venezuela, was the first to show that co-infection with *H. pylori* and intestinal helminths was associated with increased IL4 expression, together with lower degrees of inflammation and gastric atrophy, compared to mono-infected patients (25). Similarly, in a study from Colombia (a high endemic area for parasites), with virulence-associated genotypes of *H. pylori*, the rate of GCA was low, suggesting a modulating role of the immune system by helminths (26). In a third study from China, co-infection with helminths altered serological IgG responses as well as the pepsinogen I/II ratio, with attendant lower risk of gastric atrophy (27), again supporting the role of helminths in potentially explaining the enigma. In Brazil, however, this association was not found; although co-infection with parasites was as high as 15% in adults, polarization of the immune system toward a Th1 or Th2 response was independent of co-infection (28), throwing into doubt the findings of previous studies. Although these countries are similar in economic development to SSA, unfortunately no studies have been conducted in SSA. As such, only inferences can be made regarding the potential role of helminths in explaining the enigma. More recent data from SSA, however, reports that those with African ancestry demonstrate co-evolution with *H. pylori* compared to their European counterparts, in whom this appears to be maladapted (29). This finding suggests that *H. pylori* may be “less pathogenic” in this population, thus explaining the enigma possibly based on co-infection with parasites, the gastric microbiome or dietary factors.

Notwithstanding the above explanations, to date, however, this hypothesis has not been fully elucidated, with some questioning its existence (30). Detractors of this theory argue that it is not uncommon for infectious disease to present with variable clinical expressions in different regions, and that GCA would be expected in regions with longer life expectancy (which has not been the case in SSA), and higher rates of atrophic gastritis. Furthermore, they argue that rather than focus on individual populations, it is the convergence of factors (bacterium, host and environmental factors), that play a role in disease expression (30). Finally, they argue that the low rates of GCA may merely reflect selection bias of studies that include populations with limited access to endoscopy and limited data for explanations such as *H. pylori* strain and the role of parasites in modulating the immune response toward a Th2 phenotype. Hence these authors posit that rather than a hypothesis based on experimental data, this enigma is a myth. The lack of data, however, makes this assertion difficult to make, but interesting as potential for future research.

To summarize, inconsistent data is the key limiting factor in the argument for or against the enigma. However, given the high prevalence of helminth infections in SSA (28, 31) this provides a niche area of research not only in terms of understanding the pathophysiology of *H. pylori*, the enigma more specifically, but also the potential for using helminths as a therapeutic tool in preventing GCA (and potentially other gastrointestinal cancers) not just in SSA, but worldwide.

## Inflammatory bowel disease (IBD) in SSA

Inflammatory bowel disease has largely been a disease affecting high-income countries; in particular North America and Europe. Over recent years an increase in cases of newly diagnosed IBD has also been reported in North Africa, Asia, and South America (6, 7).

In contrast, apart from South Africa, IBD has been considered rare in SSA. The recent Global Burden of Disease study reported age standardized prevalence rates in SSA ranging between 9.9 and 11.2 per 100,000 population (10). These rates are in stark contrast with Westernized countries such as North America, where a prevalence of 442 per 100,000 people was reported for 2017 (10). Two systematic reviews published in 2020 identified fewer than 250 published cases of IBD in SSA, after excluding those from SA (8, 9). This paucity of cases may represent under reporting or misdiagnosis due to a high burden of infectious diseases in the sub-continent which can mimic IBD, notably Amebiasis, Schistosomiasis, Strongyloidiasis, and Intestinal Tuberculosis (32). It may also reflect a different genetic disposition or low exposure to environmental risk factors described in high income populations. In addition, some cases of IBD in SSA might remain undetected because of a lack of awareness or limitations in diagnostic and clinical capacity (32).

Low rates of IBD may, however, also be explained by the high prevalence of chronic gut infections endemic in the region which appear to attenuate the risk of developing IBD, notably *H. pylori* and parasitic infestation with helminths. Both chronic infections induce systematic immune tolerance and suppress inflammatory responses. This is consistent with the hygiene hypothesis which proposes a causal relationship between increased sanitation leading to reduced exposure to microorganisms and an increase in autoimmune disease such as IBD (33).

### *H. pylori* and IBD

In contrast to GCA several studies have shown a protective association between *H. pylori* infection and IBD. In a meta-analysis of 40 case-control studies, *H. pylori* exposure was associated with a significant reduction in the risk of developing IBD (OR 0.43, 95% CI 0.36–0.50) (34). This observation was stronger for CD than for UC. Similar findings have been reported in animal models, where *H. pylori* exposure has been associated with attenuation of experimental colitis (35, 36).

There are several biologically plausible mechanisms whereby *H. pylori* infection may protect against the development of IBD. Over millennia *H. pylori* has adapted to evade the hosts innate and adaptive immune responses, to maintain mucosal colonization. *H. pylori* infection appears to induce a blunted Th1/Th17 response with an enhanced immunosuppressive T-reg response (37) and also induces tolerogenic dendritic cells (DCs) (36).

In a meta-analysis this protective effect of *H. pylori* was restricted to CagA-positive strains, with no significant difference in the risk of IBD between *H. pylori* CagA seronegative strains, and *H. pylori* non-exposed individuals (19). The beneficial effect of CagA positivity can be at least partly explained by the increased IL10 responses inducing semi-mature DC and higher T-reg responses (38). Of interest 95% of *H. pylori* strains in a South African study were CagA positive (39).

The inverse relationship between *H. pylori* infection and the development of IBD may, however, be confounded by many other factors implicated in the hygiene hypothesis in that the more sterile environment encountered in higher income regions reduces the likelihood of *H. pylori* infection (33, 40). It is unclear if treatment for *H. pylori* infection is associated with an increase in the risk of subsequently developing IBD as the evidence is conflicting (41, 42).

### IBD and helminth infection

Soil-transmitted helminths such as *Ascaris lumbricoides*, *Trichuris trichiura*, and the hookworms *Ancylostoma duodenale* and *Necator americanus*, are endemic in SSA. These infections are transmitted by eggs present in human feces, which contaminate the soil, and as with *H. pylori* are associated with overcrowding, less industrialization, and poorer water sanitation, all of which may be protective of IBD (33, 43).

Similar to *H. pylori*, helminth infection appears to modulate the host response leading to a state of relative immune tolerance (44, 45). Infection by helminths has been shown to attenuate experimental colitis in mouse models as well as impact the composition of the gastrointestinal microbiome (46–48). There is also evidence from human studies that infections with tissue-dwelling helminths initiate expansion of T-regulatory (T_reg_) cells that suppress both Th1 and Th2 effector responses and up-regulate the release of anti-inflammatory cytokines such as IL10 and transforming growth factor (TGFβ) (49). In a study of schoolchildren in Cameroon it was shown that levels of these regulatory cytokines correlate with the intestinal worm burden and that this elevation in anti-inflammatory cytokine secretion in peripheral blood induces immunological hypo-responsiveness (49). It has been hypothesized that worm-driven up-regulation of IL10 and or TGFβ secreting T_reg_ cells, represent a parasite survival strategy to suppress an effective host immune response.

An inverse association between helminth infections and the development of IBD in SSA has been described. A South African study of 151 patients with IBD showed that childhood helminth exposure was associated with 80% risk reduction for the development of IBD compared with controls (12).

Several studies have evaluated the efficacy of the pig whipworm *Trichuris suis* for the treatment of IBD with mixed results; initial reports were encouraging, however, a meta-analysis of *T. suis* ova in human clinical trials showed no statistically significant effect (50). However, the included studies were limited by small sample size which may have masked any potential benefit. The potential therapeutic benefit of helminth infection and helminth products is being further explored (51).

## Conclusion

Gastric adenocarcinoma and IBD are common disorders globally, yet surprisingly low numbers of both disorders are reported in Sub-Saharan Africa (SSA). This may reflect under-reporting or limited access to diagnostic modalities, but an alternative explanation is the high burden of infection with gastrointestinal helminths, which may attenuate the risk of developing GCA and IBD through induction of immune tolerance. In contrast to GCA several studies have shown a protective association between *H. pylori* infection and IBD. Given the lack of high- quality data from SSA these observations require further investigation, as they may provide important insights that can be exploited for the development of diagnostic and/or therapeutic tools in the management of these conditions.

## Author contributions

Both authors contributed equally to the conception, initial, and revised drafts of the manuscript, and approved the submitted version.
